# Circ-0005105 activates COL11A1 by targeting miR-20a-3p to promote pancreatic ductal adenocarcinoma progression

**DOI:** 10.1038/s41419-021-03938-8

**Published:** 2021-06-28

**Authors:** Gang Ma, Guichen Li, Wufeng Fan, Yuanhong Xu, Shaowei Song, Kejian Guo, Zhe Liu

**Affiliations:** Department of Pancreatic-Biliary Surgery, First Hospital of China Medical University, Shenyang, China

**Keywords:** Pancreatic cancer, Cell invasion

## Abstract

Growing evidence indicates that circular RNAs (circRNAs) are closely involved in tumorigenesis, but the association between circRNAs and pancreatic ductal adenocarcinoma (PDAC) is far from clear. Here, we focused on the functional investigation of circ-0005105, a newly identified circRNA, in PDAC progression. In the present study, we assessed circ-0005105 expression in PDAC tissues and cell lines with quantitative reverse transcription–polymerase chain reaction (qRT-PCR). The biological functions of circ-0005105 in cellular proliferation and invasion were identified through gain- and loss-of-function experiments in vitro and in vivo. The interaction between circ-0005105 and the microRNA (miR)-20a-3p–COL11A1 (collagen type XI alpha 1) axis was examined using luciferase reporter and RNA immunoprecipitation assays. We found that circ-0005105 expression was upregulated in both PDAC tissues and cell lines. Higher circ-0005105 expression correlated positively with the malignant clinical phenotype and poor prognosis of patients with PDAC. Gain- and loss-of-function analysis showed that circ-0005105 facilitated both in vitro and in vivo cellular proliferation and invasion. Mechanistically, circ-000510 served as a competing endogenous RNA (ceRNA) of miR-20a-3p and indirectly modulated *COL11A1* expression, leading to activation of epithelial–mesenchymal transition (EMT). Rescue experiments suggested that the oncogenic activity of circ-0005105 was dependent on the modulation of the miR-20a-3p–COL11A1 axis. More importantly, *COL11A1* overexpression was significantly associated with poor prognosis in PDAC, and silencing *COL11A1* reduced PDAC cell tumorigenicity and metastasis. Taken together, our findings confirm for the first time that circ-0005105 has critical functions by regulating the miR-20a-3p–COL11A1 axis. In the clinic, circ-0005105 can act as a potential prognostic marker and therapeutic target in PDAC.

## Background

Globally, pancreatic ductal adenocarcinoma (PDAC) is gastrointestinal cancer with high mortality; it is known for being elusive to early detection and its unfavorable prognosis [1]. Its high rates of regional invasion and systemic reoccurrence, involving peritoneal metastasis and retroperitoneal reoccurrence, also compromise therapeutic efficacy [2]. Despite great efforts being made in terms of treatment, the 5-year overall survival (OS) rate of PDAC (about 8%) has not changed much in the last few years [3]. Therefore, it is crucial to elucidate the molecular mechanisms underlying progression and metastasis for treating PDAC.

Non-coding RNAs (ncRNAs), consisting of various RNAs such as microRNAs (miRNAs), long non-coding RNAs (lncRNAs), and circular RNAs (circRNAs), participate significantly in cellular development and pathogenesis [4]. circRNAs originate from mRNA splicing and are characterized by covalently closed continuous loops [5]. Increasing evidence suggests that circRNAs participate in various physiological and pathological processes [6]. In particular, they participate in the tumorigenesis and progression of several types of malignant tumors [7]. Recent studies have identified a number of important circRNAs in PDAC, such as circ-ASH2L [8], circ-LDLRAD3 [9], circ_0030235 [10], CircFOXK2 [11], hsa_circ_001653 [12], ciRS-7 [13], and circBFAR [14]. Nevertheless, to our best knowledge, the specific molecular mechanisms underlying how circRNAs function in PDAC progression remain largely unknown.

Recently, there has been increased interest in the regulatory mechanism reported in the competing endogenous RNA (ceRNA) hypothesis [15]. circRNAs are considered part of the ceRNA family. Prior studies have suggested that circRNAs might exert effects on the pathogenesis and development of malignancies through ceRNA regulatory mechanisms [16, 17]. For example, Li et al. found that the circRNA circZNF566 acts as a ceRNA for the miR-4738-3p–TDO2 axis and promotes hepatocellular carcinoma progression [18]. Sun et al. revealed that circRNA-100290 has a significant effect on oral cancer by sponging the miR-29 family [19]. In PDAC, the study of circRNAs is in the initial stage. Several circRNAs are involved in PDAC progression in a ceRNA-dependent manner [8–14]. Therefore, it is essential to elucidate the relationship between circRNA expression and PDAC development.

In the present study, we demonstrate the significant upregulation of circ-0005105 in PDAC tissues and its association with poor clinical prognosis. Functional experiments revealed that circ-0005105 markedly contributed to cellular proliferation, invasion, and migration in vitro and in vivo. Further study revealed that circ-0005105 promoted collagen type XI alpha 1 (*COL11A1*) expression by sponging miR-20a-3p, and activated epithelial–mesenchymal transition (EMT). Finally, rescue experiments validated the fact that circ-0005105 accelerates PDAC tumor growth and metastasis potential by targeting the miR-20a-3p–COL11A1 axis. To summarize, our findings reveal a critical role of the circ-0005105–miR-20a-3p–COL11A1 axis in mediating EMT activation in PDAC progression.

## Results

### circ-0005105 expression is upregulated in PDAC tissues and cell lines

**Fig. 1:**
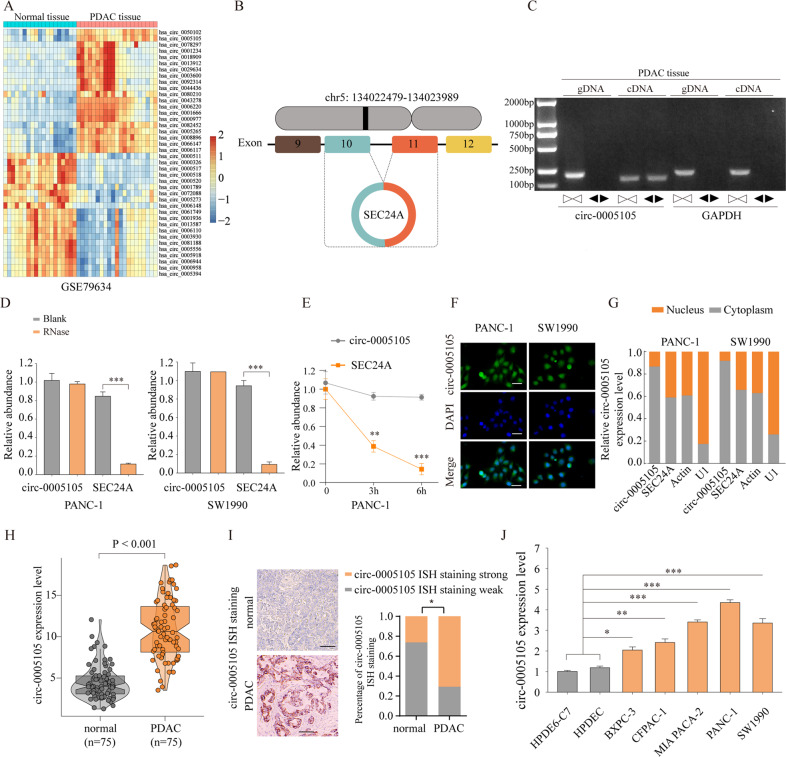
CircRNA circ-0005105 is overexpressed in PDAC tissues. **A** Heat maps of differently expressed circRNAs in PDAC tissue compared with adjacent normal tissue (GSE79634). Red indicates a higher fold-change and blue indicates a lower fold change. **B** circ-0005105 is produced at the SEC24A gene locus containing exon 9-12. **C**. RT-PCR assay with divergent or convergent primers indicated the existence of circ-0005105 in PDAC tissue. GAPDH was used as a negative control. qRT-PCR analysis of the expression of circ-0005105 after RNase R treatment (**D**) and Actinomycin D (**E**). The subcellular localization of circ-0005105 in PANC-1 and SW1990 cells were analyzed through fluorescence in situ hybridization (FISH) (**F**) and nuclear and cytoplasmic separation assay; Scale bar = 40 μm (**G**). The expression of circ-0005105 in 75 pairs of PDAC (Tumor) and adjacent normal tissues (Normal) was determined by qRT-PCR (**H**) and in situ hybridization (ISH); Scale bar = 400 μm (**I**). qPCR analysis of circ-0005105 expression in pancreatic epithelial cells (HPDE6-C7 and HPDEC) and pancreatic cancer cells (BXPC-3, CFPAC-1, MIA PACA-2, PANC-1, and SW1990). The results are presented as the mean ± SD. *P < 0.05, **P < 0.01, ***P < 0.01.

To identify the differentially expressed circRNAs in PDAC, we first analyzed public circRNA microarrays (GSE79634) obtained from the Gene Expression Omnibus (GEO) database, and found that circ-0005105 was among the most significantly differentially expressed circRNAs between tumor tissues (*n* = 10) and adjacent normal tissues (*n* = 10) (Fig. 1A). Figure 1B shows that circ-0005105 is an exon circRNA transcript from the protein-coding gene *SEC24A*, located on chromosome 5q13. Next, we detected circ-0005105 expression using the divergent primer strategy to validate the circularized junction of the circRNAs (Fig. 1C). Resistance to RNase R exonuclease and actinomycin D confirmed the circular structure of circ-0005105 (Fig. 1D, E). Additionally, we detected the subcellular localization of circ-0005105 in PANC-1 and SW1990 cells through fluorescence in situ hybridization (FISH) (Fig. 1F) and nuclear and cytoplasmic separation assay (Fig. 1G), and found that circ-0005105 was predominantly localized in the cytoplasm. Quantitative reverse transcription–polymerase chain reaction (qRT-PCR) and in situ hybridization (ISH) showed higher circ-0005105 expression in PDAC tissues than in the non-tumorous tissues (Fig. 1H, I). circ-0005105 was more significantly upregulated in pancreatic cancer cells (BxPC-3, CFPAC-1, MIA PaCa-2, PANC-1, and SW1990) compared with pancreatic epithelial cells (HPDE6-C7 and HPDEC) (Fig. 1J).

### Elevated circ-0005105 expression predicts poor clinical outcomes in PDAC

**Fig. 2:**
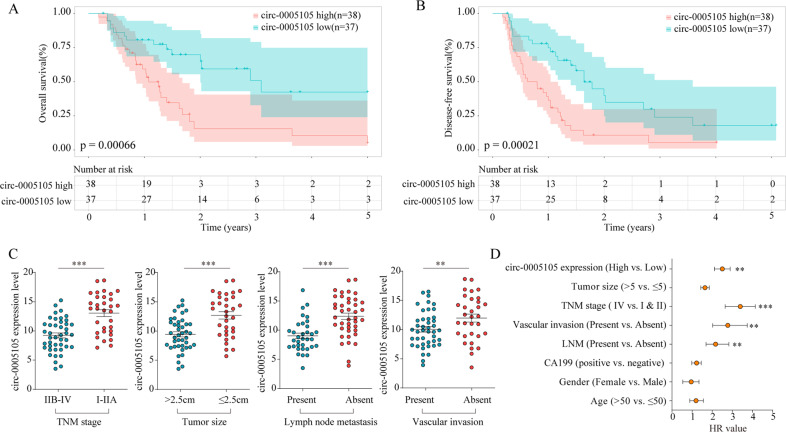
Upregulated circ-0005105 was positively correlated with poor prognosis of PDAC. Patients with higher circ-0005105 expression had a shorter overall survival time (**A**) and disease-free survival time (**B**) than those with lower circ-0005105 expression. **C** The correlation between the expression of circ-0005105 and TNM stage, tumor size, lymph node metastasis, and vascular invasion of PDAC patients. **D** The multivariate Cox regression analyses were performed to depict the correlations between the indicated clinical criteria and circ-0005105 expression level. **P < 0.01, ***P < 0.001.

To evaluate the clinical significance of circ-0005105 expression, patients with PDAC were separated into two groups based on circ-0005105 expression level, i.e., high and low. Subsequently, we examined the correlation between circ-0005105 expression and the clinicopathological attributes of PDAC: patients with high circ-0005105 expression had unfavorable overall survival (OS) (*P* < 0.001, Fig. 2A) and disease-free survival (DFS) (*P* < 0.001, Fig. 2B) than those with lower circ-0005105 expression. Moreover, high circ-0005105 levels were closely related to tumor-node-metastasis (TNM) stage (*P* = 0.018), tumor size (*P* = 0.033), lymph node metastasis (*P* = 0.018), and vascular invasion (*P* = 0.049) (Fig. 2C). Multivariate analyses demonstrated that circ-0005105 expression was an independent prognostic factor for poor outcomes in patients with PDAC (Fig. 2D). To sum up, these findings determine that elevated circ-0005105 expression is a potential prognostic indicator of unfavorable prognosis in patients with PDAC.

### circ-0005105 promotes PDAC cell proliferation, invasion, and migration

**Fig. 3:**
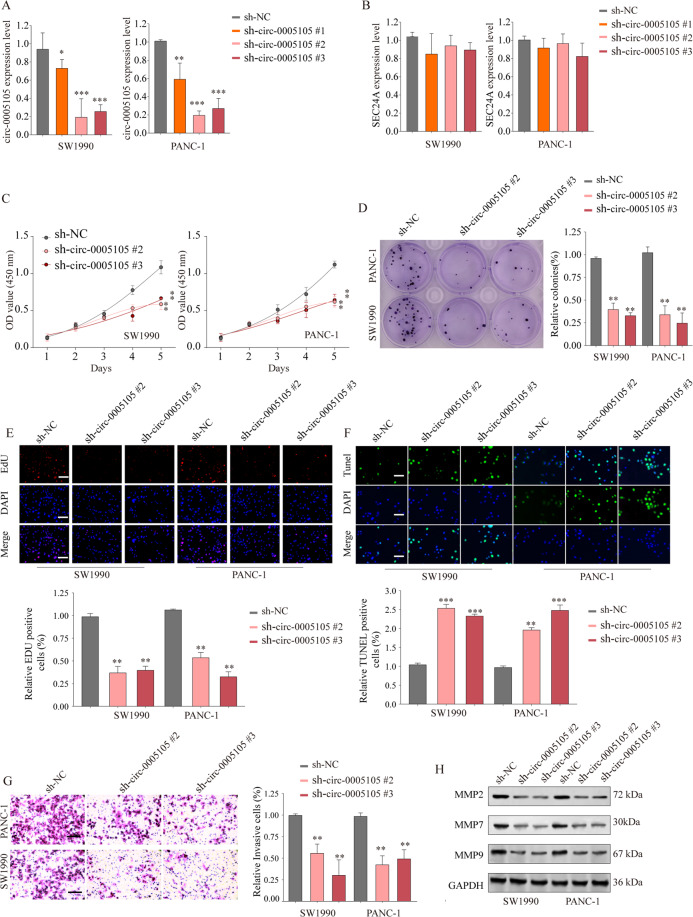
circ-0005105 knockdown inhibit PDAC cell proliferation and invasion. Pancreatic cancer cell line SW1990 or PANC-1 was transfected with negative control (shNC) or shRNA targeting circ-0005105 (sh-circ-0005105 #1/2/3). The relative expression levels of circ-0005105 (**A**) or SEC24A mRNA (**B**) were analyzed by qRT-PCR. Cell proliferation capability of SW1990 or PANC-1 cells transfected with sh-NC or sh-circ-0005105 #2/3 was determined by CCK-8 assay (**C**), colony formation assay (**F**), and EdU assay (**E**); Scale bar = 50 μm. **F** Cell apoptosis was determined by TUNEL assay; Scale bar = 50 μm. **G** Transwell experiment was performed to analyze the cell invasion capability of SW1990 or PANC-1 cells transfected with sh-NC or sh-circ-0005105 #2/3; Scale bar = 50 μm. **H** Expression levels of MMP-2, MMP-7, and MMP-9 in SW1990 or PANC-1 transfected with sh-NC or sh-circ-0005105 #2/3 were analyzed by western blot. The results are presented as the mean ± SD. *P < 0.05, **P < 0.01, ***P < 0.001.

To explore the possible functional roles of circ-0005105 in PDAC progression, endogenous circ-0005105 expression in PANC-1 and SW1990 cells was silenced with three short hairpin RNAs (shRNAs) specifically targeting circ-0005105 (sh-circ-0005105 #1–3). qRT-PCR confirmed significantly decreased circ-0005105 expression while revealing no significant difference in the expression of its host gene *SEC24A* in circ-0005105 knockdown PANC-1 and SW1990 cells as compared with the control cells (Fig. 3A, B). The Cell Counting Kit-8 (CCK-8) and colony formation assays showed that the downregulation of circ-0005105 inhibited PDAC cell growth and proliferation capabilities (Supplementary Fig. S1A; Fig. 3C, D). Moreover, circ-0005105-knockdown cells had noticeably decreased DNA synthesis as compared with the control groups (Fig. 3E). TUNEL (terminal deoxynucleotidyl transferase-mediated dUTP nick end labeling) indicated that the circ-0005105-knockdown cells had an increased apoptosis rate (Fig. 3F).

We used Transwell invasion and wound healing assays to verify whether circ-0005105 knockdown affected PDAC cell invasion and migration. circ-0005105 knockdown PANC-1 and SW1990 cells had consistently and substantially decreased invasion and migration capabilities (Supplementary Fig. S1B; Fig. 3G). Moreover, decreased metastasis-associated proteins, i.e., MMP-2, MMP-7, and MMP-9, were detected in the circ-0005105 knockdown cells (Fig. 3H). In contrast, circ-0005105 overexpression promoted PDAC cell proliferation and invasion (Supplementary Fig. S2). Collectively, these findings indicate a crucial role of circ-0005105 in facilitating PDAC cell growth and metastasis.

### Silencing circ-0005105 inhibits PDAC tumorigenesis and invasiveness in vivo

**Fig. 4:**
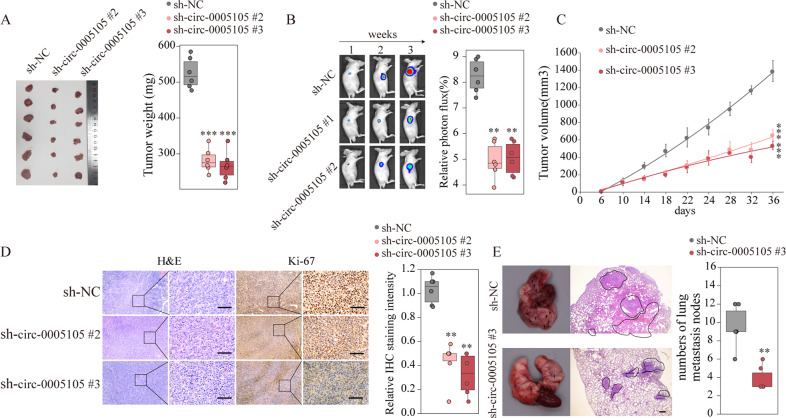
circ-0005105 promotes the PDAC tumorigenesis and metastasis in vivo. Pancreatic cancer cell PANC-1 stably transfected with negative control (sh-NC) or sh-circ-0005105 #2/3 were intravenous injected into nude mice. **A** Tumors from sh-NC or sh-circ-0005105 #2/3 group were isolated from nude mice and tumor weight was examined. **B** Representative bioluminescent photos were recorded at 1, 3, 5 weeks post injection. Relative luciferase activity was analyzed in sh-NC or sh-circ-0005105 #2/3 group. **C** Tumor volume was measured and determined at indicated time points. **D** Representative H&E staining and immunohistochemical staining image of Ki-67 were acquired on tumor sections from sh-NC or sh-circ-0005105 #2/3 group. Relative Ki-67 staining intensity in sh-NC or sh-circ-0005105 #2/3 group was analyzed. Scale bar = 200 μm. **E** Representative results of gross and H&E staining of metastatic lung nodules in different groups. Scale bars, 500 μm. The results are presented as the mean ± SD for each group (*n* = 6). *P < 0.05, **P < 0.01, ***P < 0.001.

Next, we investigated whether circ-0005105 could exert a promoting effect on tumorigenesis and metastasis in vivo. Stable circ-0005105 knockdown PANC-1 cells or control PANC-1 cells were orthotopically injected subcutaneously into immunodeficient nude mice. The mice injected with the circ-0005105 knockdown cells had significantly lower average xenograft tumor weights (Fig. 4A). Consistently, the bioluminescence signal of the tumors decreased with circ-0005105 suppression, suggesting that stable silencing of circ-0005105 efficiently suppressed PDAC cell growth in vivo (Fig. 4B, C). In addition, immunohistochemical (IHC) analysis showed markedly decreased expression of the tumor proliferation marker Ki-67 in xenograft tumor tissues from the circ-0005105 knockdown group compared with that from the negative control group (Fig. 4D). Next, the effect of circ-0005105 on pancreatic cancer metastasis was examined using a nude mouse model of pulmonary metastasis, which was established by intravenous administration of PANC-1 cells with or without a stable circ-0005105 knockdown. Hematoxylin–eosin (H&E) staining performed after 8 weeks showed markedly fewer and smaller metastatic pulmonary nodules in the circ-0005105 knockdown group (Fig. 4E). Taken together, these results indicate that circ-0005105 can advance PDAC cellular proliferation and metastasis in vivo, which is consistent with the in vitro results.

### circ-0005105 functions as a sponge for miR-20-3p, and *COL11A1* is a direct target of miR-20-3p

**Fig. 5:**
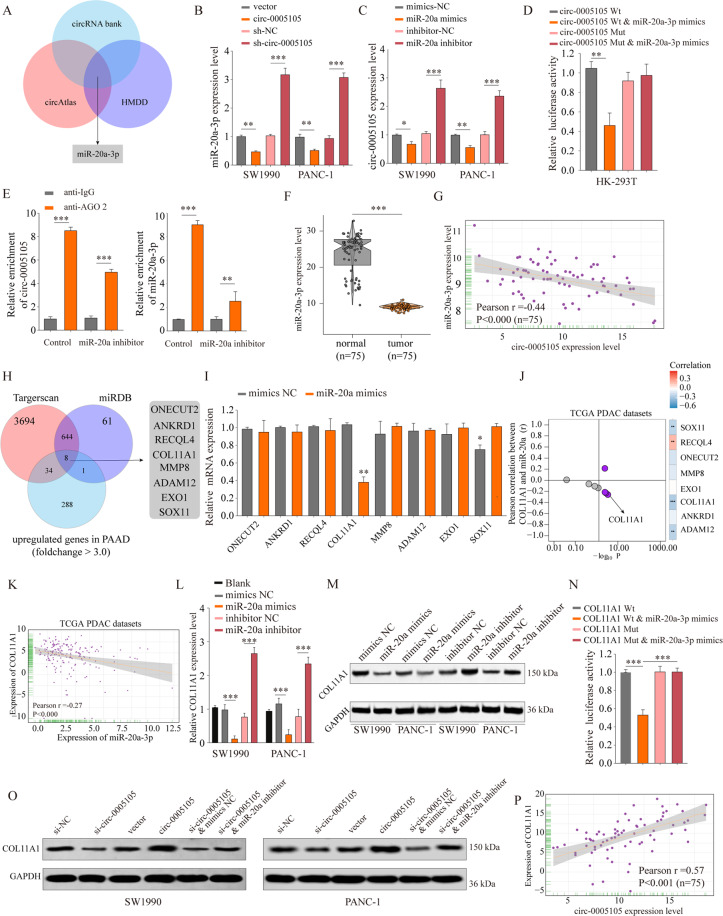
circ-0005105 acts as a sponge for miR-20a-3p, and COL11A1 is a direct target of miR-20a-3p. **A** The intersection of miRNAs targeted circ-0005105 predicted by circRNA bank, circAtlas, and miRNAs related to pancreatic cancer reported in the HMDD database is shown via Venn diagram. The relative expression of miR-20a-3p (**B**) and circ-0005105 (**C**) in SW1990 or PANC-1 cells was analyzed by qRT-PCR after indicated transfection. **D** Cells were transfected with a luciferase reporter plasmid containing wide-type (circ-0005105 Wt) or mutated circ-0005105 (circ-0005105 Mut), together with or without miR-20a-3p mimics. The relative luciferase activity was analyzed. **E** Immunoprecipitation of AGO2 (control, anti-IgG) in miR-20a-3p inhibitor or miR-inhibitor control transfected PANC-1 cells were performed. **F** The expression levels of miR-20a-3p in 75 paired pancreatic cancer tissues and non-tumor tissues were analyzed by qRT-PCR. **G** Pearson analysis of the correlation between miR-20a-3p expression and circ-0005105 expression in pancreatic cancer tissues. **H** The intersection of mRNAs potential targeting by miR-20a-3p predicted by Targetscan, miRDB, and mRNAs upregulated in pancreatic cancer reported in TCGA PAAD database is shown via Venn diagram. **I** Expression of candidate mRNAs were detected under treatment of miR-20a-3p mimics by qRT-PCR in PANC-1 cells. **J, K** Pearson analysis of the correlation between miR-20a-3p expression and candidate mRNAs expression in TCGA dataset. purple indicates *p* < 0.05. **L, M** The relative expression of COL11A1 in SW1990 or PANC-1 cells was analyzed by qRT-PCR and western blot after indicated transfection. **N** Cells were transfected with luciferase reporter plasmid containing wide-type (COL11A1 Wt) or mutated COL11A1 (COL11A1 Mut), together with or without miR-20a-3p mimics. The relative luciferase activity was analyzed. **O** The relative expression of COL11A1 in SW1990 or PANC-1 cells was analyzed by western blot after indicated transfection. **P** Pearson analysis of the correlation between COL11A1 expression and circ-0005105 expression in pancreatic cancer tissues. The results are presented as the mean ± SD for each group (*n* = 6). *P < 0.05, **P < 0.01, ***P < 0.001.

circRNAs are considered miRNA sponges, and contribute to cancer progression [20]. To investigate the underlying molecular mechanisms of circ-0005105 in PDAC progression, we screened the target miRNAs of circ-0005105 via bioinformatics analysis. The miRNA targets of circ-0005105 were predicted using Circbank (http://www.circbank.cn) and circAtlas (http://circatlas.biols.ac.cn), and the miRNAs associated with pancreatic cancer recorded in the Human MicroRNA Disease Database (HMDD, http://www.cuilab.cn/hmdd) were identified. Figure 5A shows that miR-20a-3p was the only result when the above two approaches were overlapped. qRT-PCR revealed that circ-0005105 overexpression decreased miR-20a-3p expression significantly, while circ-0005105 knockdown enhanced it (Fig. 5B). Likewise, miR-20a-3p overexpression significantly decreased circ-0005105 expression in PANC-1 or SW1990 cells, while miR-20a-3p suppression increased it (Fig. 5C). The dual-luciferase reporter assay indicated that the circ-0005105 wild-type (WT) and miR-20a-3p groups had significantly decreased relative luciferase activity in comparison with the circ-0005105 mutant (Mut) and miR-20a-3p groups (Fig. 5D). Furthermore, AGO2 immunoprecipitation qPCR showed that, in comparison with the anti-IgG (immunoglobulin G) group, the anti-AGO2 group had substantially elevated relative abundance of circ-0005105 and miR-20a, while circ-0005105 and miR-20a-3p immunoprecipitation with AGO2 was decreased after miR-20a-3p silencing (Fig. 5E). Furthermore, PDAC tissues had substantially reduced miR-20a-3p expression (Fig. 5F). Pearson analysis showed a negative connection between circ-0005105 and miR-20a-3p expression in the PDAC tissues (Fig. 5G). Taken together, these findings reveal an association of miR-20a-3p with circ-0005105.

Subsequently, the mRNA targets of miR-20a-3p were predicted using TargetScan (http://www.targetscan.org) and miRDB (http://mirdb.org), and the miRNAs downregulated in PDAC tissues were screened based on The Cancer Genome Atlas (TCGA) database analysis. Eight candidate mRNAs (*ONECUT2*, *ANKRD1*, *RECQL4*, *COL11A1*, *MMP8*, *ADAM12*, *EXO1*, *SOX11*) were selected (Fig. 5H). Validation experiments revealed that miR-20a-3p overexpression significantly decreased *COL11A1* mRNA levels (Fig. 5I). Pearson analysis showed a significant negative correlation between *COL11A1* and miR-20a-3p expression (Fig. 5J, K). Moreover, miR-20a-3p knockdown significantly increased *COL11A1* expression in PDAC cells, whereas enforced expression of miR-20a-3p reduced *COL11A1* expression significantly (Fig. 5L, M). Additionally, the dual-reporter luciferase assays indicated that miR-20a-3p mimic reduced COL11A1-Wt luciferase activity significantly, but not that of COL11A1-Mut (Fig. 5N). Rescue experiments revealed that miR-20a-3p inhibitor partly reversed the inhibitory effect of circ-0005105 silencing on *COL11A1* expression, which was confirmed by qRT-PCR and western blotting (Fig. 5O). Pearson analysis showed a positive connection between circ-0005105 and *COL11A1* expression (Fig. 5P; Supplementary Fig. S3). To summarize, these findings suggest that circ-0005105 sponges miR-20a-3p, which subsequently enhances *COL11A1* translation.

### Aberrant *COL11A1* expression correlates positively with malignant behaviors of PDAC

**Fig. 6:**
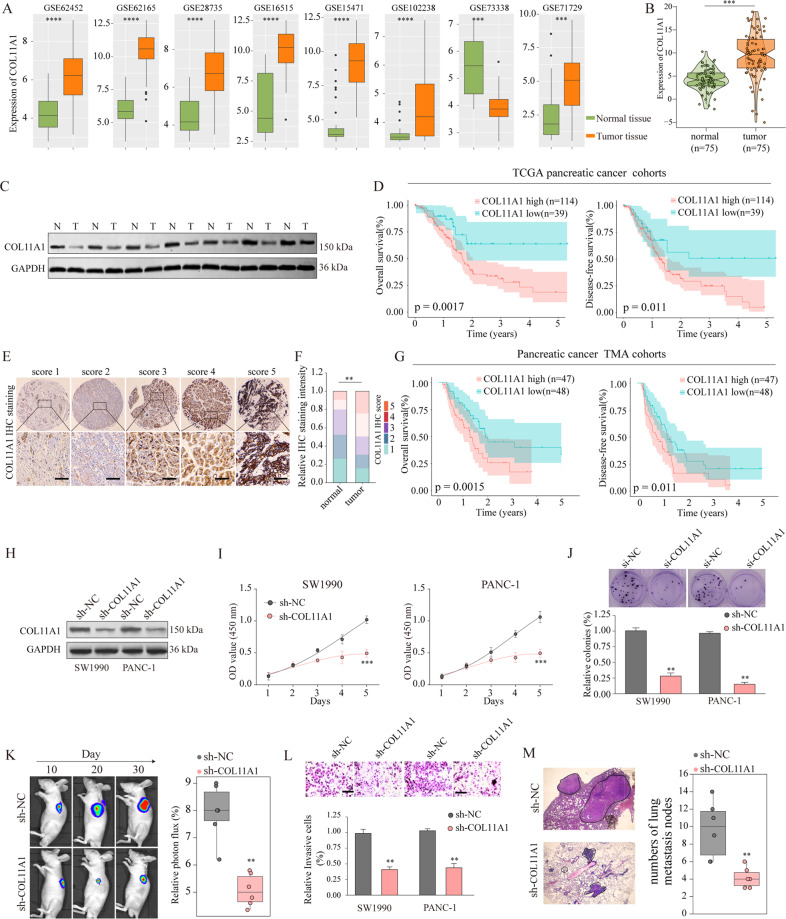
circ-0005105 acts as a sponge for miR-20a-3p, and COL11A1 is a direct target of miR-20a-3p. COL11A1 was upregulated in pancreatic cancer tissues and overexpression of COL11A1 promotes cancer progression. COL11A1 expression in pancreatic cancer and adjacent normal tissues were analyzed in multiple Gene Expression Omnibus (GEO) datasets (**A**) and our own cohort (**B**). **C** Expression of COL11A1 in pancreatic cancer tissues and adjacent non-tumor tissues was detected by western blot. **D** Kaplan–meier analysis was used to analyze the relationship between COL11A1 expression and prognosis of pancreatic cancer in TCGA dataset. **E, F** Representative IHC staining of COL11A1 and the distribution of COL11A1 staining intensity in pancreatic cancer tissues and non-tumor control tissues. Scale bars, 200 μm. **G** Kaplan–Meier survival analysis of survival rate between pancreatic cancer patients with low or high COL11A1 expression. **H** Western blot analysis of COL11A1 expression in SW1990 or PANC-1 cells transfected with negative control (sh-NC), or lentivirus targeting COL11A1 (sh- COL11A1). Cell proliferation capability of SW1990 or PANC-1 cells transfected with sh-NC or sh- COL11A1 was determined by CCK-8 assay (**I**), colony formation assay (**J**) in vitro, and tumor xenografts experiment (**K**) in vivo. Cell proliferation capability of SW1990 or PANC-1 cells transfected with sh-NC or sh- COL11A1 was determined by CCK-8 assay (**I**), colony formation assay (**J**) in vitro, and tumor xenografts experiment (**K**) in vivo. Metastasis capability of pancreatic cancer cells after COL11A1 silencing was determined by transwell assay (**L**) in vitro, and intravenous pulmonary metastasis experiment (**M**) in vivo. The results are presented as the mean ± SD for each group (*n* = 6). *P < 0.05, **P < 0.01, ***P < 0.001.

To determine the potential functional role of COL11A1 in PDAC progression, we explored the *COL11A1* expression levels in the GEO database and clinical specimens. Figure 6A–C show that all eight GEO cohorts had significantly higher *COL11A1* expression in PDAC tissues as compared with the control, which was confirmed using our own cohort. In agreement with the above qRT-PCR observations, western blotting confirmed that COL11A1 protein levels were significantly upregulated in PDAC tissues (Fig. 6C). In addition, there was a significant positive correlation between COL11A1 and tumor proliferation markers (Ki-67 and PCNA) expression levels (Supplementary Fig. S4). Moreover, prognosis analysis revealed that patients with high *COL11A1* expression had shorter OS and DFS (Fig. 6D).

Subsequently, we used a pancreatic cancer tissue microarray (*n* = 95) for determining the association of *COL11A1* expression with clinicopathological characteristics. *COL11A1* expression levels were higher in PDAC tissues than in the non-tumor counterparts (Fig. 6E, F). Moreover, upregulated *COL11A1* expression was firmly linked to malignant clinical phenotypes (Supplementary Fig. S5). Furthermore, there was a positive link between high *COL11A1* expression and worse OS (*P* = 0.0015) and DFS (*P* = 0.0.011) (Fig. 6G). Functionally, *COL11A1* silencing attenuated PDAC cellular proliferation (Fig. 6H–K; Supplementary Fig. S6A–C) and metastatic capabilities (Fig. 6L, M; Supplementary Fig. S6D) in vitro and in vivo. Taken together, the results suggest that COL11A1 is a crucial contributor to PDAC tumorigenesis and malignancy.

### circ-0005105 activates EMT by regulating the miR-20a-3p–COL11A1 axis

**Fig. 7:**
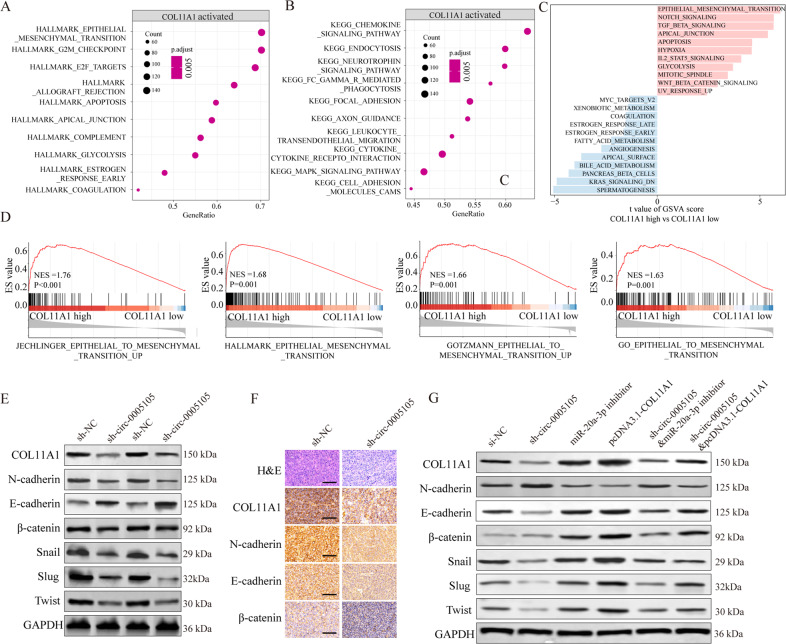
circ-0005105/miR-20a-3p/COL11A1 axis activates EMT. **A, B** KEGG pathway enrichment analysis of COL11A1 related pathways in TCGA PAAD dataset. **C** Gene set variation analysis (GSVA) and (**D**)The Gene Set Enrichment Analysis (GSEA) of the relationship between the expression level of COL11A1 and EMT-related gene signatures in the TCGA PAAD dataset. **E, F** Expression change of EMT-related proteins (N-cadherin, E-cadherin, β-catenin, Snail, Slug, and Twist) in pancreatic cells and xenograft mouse models bearing tumors under condition of COL11A1 silencing. **G** Expression change of EMT-related proteins (N-cadherin, E-cadherin, β-catenin, Snail, Slug, and Twist) in pancreatic cells after indicated transfection. The results are presented as the mean ± SD for each group. *P < 0.05, **P < 0.01, ***P < 0.001.

We explored the underlying mechanism by which circ-0005105 contributes to PDAC progression. We performed KEGG (Kyoto Encyclopedia of Genes and Genomes) analysis and gene set variation analysis (GSVA) to explore the *COL11A1*-related downstream pathways based on TCGA dataset. We found that EMT activation was positively related to *COL11A1* overexpression (Fig. 7A–C). Gene set enrichment analysis (GSEA) confirmed the significant activation of the EMT, cell adhesion molecules, and focal adhesion pathways in *COL11A1*-high expression PDAC tissues, indicating that EMT may account for the oncogenic role of *COL11A1* in PDAC (Fig. 7D).

We therefore examined whether circ-0005105 modulated EMT by regulating the miR-20a-3p–COL11A1 axis. Western blotting showed that silencing circ-0005105 resulted in the reduction of various mesenchymal factors (N-cadherin, β-catenin, Snail, Slug, and Twist) and a substantial increase in the epithelial marker E-cadherin as compared with the controls (Fig. 7E). Consistently, IHC staining of xenograft tumor tissues confirmed the inhibitory effect on EMT caused by circ-0005105 silencing (Fig. 7F). Additionally, rescue experiments suggested that co-transfection of miR-20a-3p inhibitor or pcDNA3.1-COL11A1 plasmid significantly reversed the inhibitory effect on EMT induced by circ-0005105 silencing (Fig. 7G). Collectively, our results reveal that circ-0005105 activates EMT by regulating the miR-20a-3p–COL11A1 axis in PDAC cells.

### The oncogenic role of circ-0005105 is partly dependent on the miR-20a-3p–COL11A1 axis

**Fig. 8:**
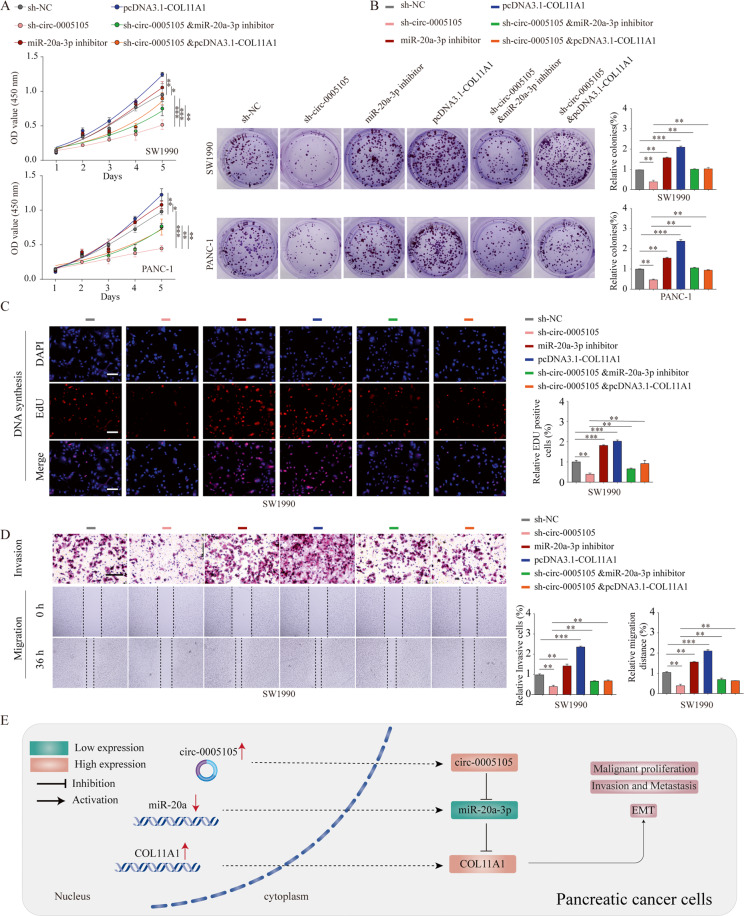
The oncogenic circ-0005105/ miR-20a-3p/COL11A1 axis in pancreatic cancer cells. **A** The CCK-8 assays, and colony formation assays (**B**), EdU assays (**C**), and transwell assay (**D**) were used to evaluate the cell growth or invasiveness after transfection with sh-NC, sh-circ-0005105, miR-20a-3p inhibitor, pcDNA3.1-COL11A1, or co-transfected with sh-circ-0005105 and miR-20a-3p inhibitor or pcDNA3.1-COL11A1 in pancreatic cancer cells. A mechanism diagram depicting that the circ-0005105/ miR-20a-3p/COL11A1 axis affects the progression of pancreatic cancer through activating EMT. The results are presented as the mean ± SD for each group. *P < 0.05, **P < 0.01, ***P < 0.001.

Finally, we verified whether the oncogenic activity of circ-0005105 in PDAC was reliant on the miR-20a-3p–COL11A1 axis. The CCK-8, EdU (5-ethynyl-2-deoxyuridine), and colony formation assays revealed that co-transfection of miR-20a-3p inhibitor or pcDNA3.1-COL11A1 plasmid significantly reversed the inhibitory effect induced by circ-0005105 silencing (Fig. 8A–C). Similarly, the decreased number of invasive cells, which was induced by circ-0005105 silencing, was reversed following miR-20a-3p downregulation or *COL11A1* upregulation (Fig. 8D). These findings indicate that circ-0005105 promotes PDAC cell proliferation and invasion, at least in part, by reducing miR-20a-3p expression and enhancing *COL11A1* expression.

## Discussion

Despite the introduction of multimodal therapies, PDAC remains one of the malignancies with the highest mortality [2, 21]. Increasing evidence suggests that circRNAs contribute crucially in modulating the pathogenesis and advancement of various types of cancer, and partially account for tumor development and invasion [22–24]. circRNA dysregulation contributes to increased cellular growth, invasion, or angiogenesis, and reduces apoptosis or dedifferentiation, ultimately resulting in tumorigenesis [17]. These features of circRNAs indicate that molecular and pathological characterization of circRNAs would be vital in improving the prognosis for cancer. Nevertheless, the specific roles of circRNAs and their correlation with the prognosis of patients with cancer remain largely unclear, particularly in the case of PDAC.

In the present study, circ-0005105 was identified as a circRNA of undetermined activity and was highly expressed in PDAC. Further investigation found that increased circ-0005105 expression was closely related with advanced TNM stage and poor prognosis. Gain- and loss-of-function experiments revealed that circ-0005105 promoted cell proliferation and invasion potential in vitro and in vivo. Therefore, we verified the oncogenic effect of circ-0005105 on PDAC. In fact, many circRNAs were previously identified as being highly dysregulated in pancreatic cancer and exhibiting oncogene or tumor-suppressing effects. Circ-ASH2L, circBFAR, ciRS-7, hsa_circ_001653, CircFOXK2, circ-LDLRAD3, and circ_0030235 act as oncogenes in pancreatic cancer [8–14]. Taken together, this collective evidence indicates that circRNAs play a crucial role in the progression of pancreatic cancer. Nevertheless, the overall function and mechanisms of circ-0005105 in PDAC are largely unexplored.

Prior studies have shown that circRNAs of exonic origin usually localize in the cytoplasm, and sometimes act as miRNA sponges [24]. Here, we reveal that circ-0005105 can bind to miR-20a-3p. To date, there have been no reports on the potential functional roles of miR-20a-3p in cancer. We discovered significant upregulation of miR-20a-3p in PDAC samples, and COL11A1 was a functional target of miR-20a-3p. Moreover, COL11A1 expression could be modulated by miR-20a-3p, which was negated effectively by circ-0005105. These findings suggest that circ-0005105 may prevent COL11A1 from miR-20a-3p–induced degradation via the ceRNA network. Similarly, several circRNAs are involved in pancreatic cancer progression in a ceRNA-dependent manner. For example, Wong and colleagues revealed that CircFOXK2 promotes PDAC progression by sponging miR-942 [11]. Liu et al. reported that ciRS-7 is involved in the proliferative and metastatic promotion of pancreatic cancer by targeting miR-7–involving EGFR–STAT3 signaling [13].

COL11A1, a collagen subtype critical for collagen fiber assembly, is upregulated in various types of cancer. Wu et al. have shown that COL11A1 overexpression in ovarian cancer promotes tumor progression [25]. Schmalbach et al. demonstrated that COL11A1 promotes proliferation and metastasis in neck squamous cell cancer [26]. Tessa et al. isolated stroma, tumor, and bulk samples using laser-capture microdissected (LCM) PDAC samples and reported COL11A1 as a poor prognostic stromal marker in PDAC [27]. Consistent with these reports, we validated the upregulated expression pattern of COL11A1 at both mRNA and protein level in several independent pancreatic cancer cohorts, and found that high COL11A1 expression was significantly related to malignant clinical phenotype and unfavorable prognosis for pancreatic cancer. Additionally, subsequent functional studies showed that *COL11A1* knockdown substantially reduced cell proliferative and invasive capabilities in vitro and in vivo. Most importantly, rescue experiments suggested that the oncogenic activity of circ-0005105 is dependent on the modulation of the miR-20a-3p–COL11A1 axis.

Subsequently, we performed bioinformatics analysis of the COL11A1-associated signal pathways, eventually focusing on the EMT pathway [28]. EMT is continuously activated and highly involved in invasion and metastasis in pancreatic cancer [29]. As COL11A1 is key collagen, it is unsurprising that it plays a crucial role in regulating EMT. Multiple collagens have been linked to cancer progression and chemoresistance, including collagen type I, III, V, VI, and XI [30, 31]. However, their effects on pancreatic cancer have not been explored. Here, for the first time, we observed that circ-0005105 knockdown suppresses the EMT phenotype of PDAC cells, while rescue experiments suggested that co-transfection of miR-20a-3p inhibitor or COL11A1 plasmid partially attenuates the inhibitory effect on EMT induced by circ-0005105 silencing. The results suggest that circ-0005105 promotes growth and metastasis, acting as a sponge of miR-20a-3p and subsequently upregulating its target *COL11A1* to exert its oncogenic function in PDAC (Fig. 8E).

## Conclusions

This study reveals that circ-0005105 is upregulated in PDAC tissues, and functions as a possible prognostic indicator for patients with PDAC. Mechanistically, circ-0005105 acts as a ceRNA, regulating *COL11A1* expression by decoying miR-20a-3p. Moreover, circ-0005105 advances PDAC tumorigenesis and metastasis partly by regulating circ-0005105–miR-20a-3p–COL11A1-mediated EMT. Overall, this study reveals a novel biomarker panel consisting of the circ-0005105–miR-20a-3p–COL11A1 axis, which is critical for PDAC progression, and which could be a novel therapeutic target for PDAC.

## Methods

### Public data resource and processing

Gene expression data for eight human pancreatic cancer cohorts and corresponding clinical information were obtained from the Gene Expression Omnibus (GEO; GSE62452, GSE62165, GSE28735, GSE16515, GSE15471, GSE102238, GSE73338, and GSE71729) and the Cancer Genome Atlas (TCGA) database. All data were downloaded and processed through the R software (version 3.6.1).

### Patient samples and tissue microarray (TMA)

Two independent cohorts were used in present study: (1) TMA cohort containing a total of 95 paraffin embedded pancreatic cancer specimens and nearby nontumorous tissues were purchased from Outdo Biotech (Shanghai, China) (Outdo cohort); (2) 75 pairs of fresh frozen pancreatic cancer tissue and nontumorous tissues were collected from Department of Pancreatic-Biliary Surgery, First Hospital of China Medical University. Approval of this study was gained from the Institutional Review Board of First Hospital of China Medical University. This study obtained written informed consents from all enrolled patients and was carried out in accordance with the Helsinki Declaration of 1975.

### Cell culture and transfection

Human pancreatic epithelial cells (HPDE6-C7 and HPDEC) were procured from Cell Biology,

Chinese Academy of Sciences (Shanghai, China). Pancreatic cancer cells (BXPC-3, CFPAC-1, MIA PACA-2, PANC-1, and SW1990) were procured from American Type Culture Collection (ATCC) (Manassas, VA, USA). Cells were cultured in RPMI-1640 medium (Invitrogen, CA) containing 10% fetal bovine serum (Gibco, CA) in a humidified atmosphere with 5% CO_2_ at 37 °C. Human short hairpin RNA (shRNA) constructs against circ-0005105 or COL11A1 were procured from OBiO Tech. (Shanghai, China). miR-20a-3p mimics or inhibitors were synthesized by Hanbio Technology (Shanghai, China). Cell transfection was performed using Lipofectamine 2000 (Invitrogen, CA).

### Quantitative real-time PCR (qRT-PCR) assay

Total RNA was extracted with Trizol (Invitrogen, USA), and reverse-transcribed into cDNAs by reverse transcription kit (Invitrogen, USA). qRT-PCR reaction was carried out in 7500 Real-Time PCR system (Carlsbad, CA, USA) using SYBR Green PCR Master Mix (Toyobo, Osaka, Japan). The primer sequences used were shown in Additional file 2: Table S1.

### Western blotting analysis

Tissues and cells were lysed in RIPA buffer (Solarbio, China) with a cocktail inhibitor (Beyotime, China). Protein lysates were subjected to SDS-PAGE and transfer onto PVDF membranes (Millipore, USA) was then carried out. Membrane blocking conducted, followed by incubation overnight using primary antibodies at 4 °C. Antibodies used in this study were shown in Additional file 3: Table S2. After being incubated using secondary antibodies, membranes were examined with Odyssey® CLx equipment (LI-COR, USA).

### Luciferase activity assay and RNA immunoprecipitation (RIP)

The 3′-UTR fragment of COL11A1 and circ-0005105 incorporating proposed wild-type (WT) or mutated (MUT) miR-20a-3p binding sequences were integrated onto psiCHECK-2 vector (Promega, USA) with firefly luciferase reporter. Cell were harvested at 48 h posttransfection and the Luciferase activity was detected with Dual Luciferase Reporter Assay System (Promega, USA). Interaction of miR-20a-3p with COL11A1 was detected by performing a RIP assay using anti-AGO2 and IgG antibodies. Co-precipitated RNAs were subjected to qRT-PCR detection.

### Immunohistochemical (IHC) staining

IHC staining procedures for paraffin-embedded tissue sections were performed as previously specified. For COL11A1 IHC analysis in TMA, COL11A1 staining was rated as score1/2/3/4/5 based on the positive-staining proportion and staining intensity.

### Cell proliferation assay

Cells were plated in 96-well-plates (5 × 10^3^ cells per well). Transfected cells were cultured for 0, 24, 48, 72, and 96 h, respectively, to perform CCK-8 detection. Afterward, 10 μl CCK-8 (Beyotime Biotechnology, China) was added in each well and cells were cultured for further 2 h. Absorbance (450 nm) was measured using a microplate reader (BioTek, USA). Each group had five replicates.

### Invasion assay

Transwell invasion assay was performed using Matrigel pre-coated transwell chambers (Corning, USA) in 24-well-plates. Cells were suspended in the serum-free medium. The lower chamber was added with 800 μl cultural medium containing 10% FBS. Cells were incubated with 5% CO_2_ in saturated humidity for 48 h. Subsequently, transwell chambers were washed with PBS twice and invaded cells were fixed with 4% paraformaldehyde. Crystal violet (0.4%) was used for staining for 5 min. Stained cells were visualized and counted in 5 random fields.

### Wound healing assay

Cells were cultured with serum free medium, then scratched using a 200 μl pipette tip, followed by once serum-free medium washing to remove cell debris. After observation and taking pictures (0 h), cells were cultured using the serum-free medium with 5% CO_2_ for 48 h. Subsequently, cells were photographed again (36 h) and migration distance was analyzed.

### Tumor formation assay and lung metastasis assay in a nude mouse model

For tumor formation assay, BALB/c mice (4–6 weeks old) were procured from the Gempharmatech Laboratory Animal Center (Nanjing, China). Then the cells were resuspended in 50 μL phosphate buffer saline and subcutaneously injected into each mouse. Five days after injection, tumor growth was monitored every four days. Tumor growth was followed up by visualizing tumor weight and recorded by IVIS^@^ Lumina II system (Caliper Life Sciences, USA). For pulmonary metastasis assay, BALB/c mice (4–6 weeks) were used for model construction. Cell suspension was performed in 0.1 ml PBS, followed by lateral tail vein administration into mice. Following sacrificing of mice (after 6 weeks), pulmonary metastases were detected by hematein eosin staining. Animals were operated in line with experimental animal care and use guidelines, and the experimental approval was obtained from the institutional ethics guidelines for our hospital.

### Statistical analysis

All results were collected and calculated on Graphpad prism 8.0. Statistical difference between 2 groups was tested by student *t*-test and that between multiple groups was tested by one-way or two-way analysis of variance. Comparison between two means within multiple groups was determined by Tukey’s test. Pearson method was used for the analysis of correlation. Survival analyses were conducted using the Kaplan-Meier method, and the comparison was performed using the log-rank test. Cox proportional hazards regression models were adopted for the univariate and multivariate analyses. A *P* < 0.05 was deemed to be statistically different.

## Supplementary information

Supplementary Figure Legends

supplementary table 1

supplementary table 2

Supplementary Figure S1

Supplementary Figure S2

Supplementary Figure S3

Supplementary Figure S4

Supplementary Figure S5

Supplementary Figure S6

Supplementary Figure S7

Supplementary Figure S8

## Data Availability

The datasets used and/or analyzed during the current study are available from the corresponding author on reasonable request.
