# Supplementary material for: Circ-0005105 activates COL11A1 by targeting miR-20a-3p to promote pancreatic ductal adenocarcinoma progression
Source: Cell Death Dis. 2021 Jun 28;12(7):656. doi: 10.1038/s41419-021-03938-8 (PMC8239051; doi:10.1038/s41419-021-03938-8)
Supplement: Supplementary file 1 — Supplementary Figure Legends [file 41419_2021_3938_MOESM1_ESM.docx]

Supplementary Figure legends

**Supplementary Figure S1.** circ-0005105 knockdown inhibit pancreatic cancer cell proliferation and invasion. (**A**) Representative images of SW1990 or PANC-1 cells after transfection with negative control (shNC) or shRNA targeting circ-0005105 (sh-circ-0005105 #1/2/3). (B) Micrographs of SW1990 or PANC-1 cells at 0 and 36 hours after wounding. The results are presented as the mean ± SD. **P <0.01, ***P <0.001.

**Supplementary Figure S2.** circ-0005105 overexpression promotes pancreatic cancer cell proliferation and invasion. Pancreatic cancer cell line SW1990 or PANC-1 was transfected with negative control plasmid (vector) or pcDNA3.1-circ-0005105 plasmid (circ-0005105). (A) The relative expression levels of circ-0005105 were analyzed by qRT-PCR. Cell proliferation capability of SW1990 or PANC-1 cells transfected with vector or circ-0005105 was determined by CCK-8 assay (B), colony formation assay (C) and EdU assay (D); Scale bar = 50 μm. (E) Transwell experiment was performed to analyze the cell invasion capability of SW1990 or PANC-1 cells transfected with vector or circ-0005105; Scale bar = 50 μm. The results are presented as the mean ± SD. *P <0.05, **P <0.01, ***P <0.001.

**Supplementary Figure S3.** (A) qPCR analysis of miR-20a-3p expression in pancreatic epithelial cells (HPDE6-C7 and HPDEC) and pancreatic cancer cells (BXPC-3, CFPAC-1, MIA PACA-2, PANC-1 and SW1990). (B) Pearson analysis of the correlation between miR-20a-3p expression and circ-0005105 expression in PADC cell lines. The results are presented as the mean ± SD. *P <0.05

**Supplementary Figure S4.** Representative IHC staining of COL11A1 and quantification of COL11A1 staining scores in PDAC patients from TMA cohort with high or low miR-20a-3p expression. Scale bars, 50 μm. ***P < 0.001

**Supplementary Figure S5.** (A, B) Pearson analysis of the correlation between COL11A1 expression and Ki-67 and PCNA expression in TCGA Pan-cancer dataset. purple indicates p<0.05. (C, D) Pearson analysis of the correlation between COL11A1 expression and Ki-67 and PCNA expression in TCGA PDAC dataset. purple indicates p<0.05.

**Supplementary Figure S6.** The correlation between the expression levels of COL11A1 with lymph node metastasis (A), TNM stage (B), tumor size (C), or vascular invasion (D).

**Supplementary Figure S7.** COL11A1 knockdown inhibit pancreatic cancer cell proliferation and invasion. Pancreatic cancer cell line SW1990 or PANC-1 was transfected with negative control (sh-NC) or shRNA targeting COL11A1 (sh-COL11A1). Cell proliferation capability of SW1990 or PANC-1 cells transfected with sh-NC or sh-COL11A1 was determined by EdU assay (A); Scale bar = 50 μm. (B) Cell apoptosis was determined by TUNEL assay; Scale bar = 50 μm. (C) Representative H&E staining and IHC staining image of Ki-67 and COL11A1 were acquired on tumor sections from sh-NC or sh-COL11A1 group. Relative Ki-67 staining intensity in sh-NC or sh-COL11A1 group was analyzed. Scale bar = 200 μm. (D) Micrographs of SW1990 or PANC-1 cells at 0 and 36 hours after wounding. The results are presented as the mean ± SD. *P <0.05, **P <0.01, ***P <0.001.

**Supplementary Figure S8.** Uncropped western blot scans with marker size indications
