# Supplementary material for: Circ-0005105 activates COL11A1 by targeting miR-20a-3p to promote pancreatic ductal adenocarcinoma progression
Source: Cell Death Dis. 2021 Jun 28;12(7):656. doi: 10.1038/s41419-021-03938-8 (PMC8239051; doi:10.1038/s41419-021-03938-8)
Supplement: Supplementary file 2 — supplementary table 1 [file 41419_2021_3938_MOESM2_ESM.pdf]

Supplementary Table S2. Primer sequence used in this study

Name	Direction	Primer (5'-3')
circ-0005105	Forward	5'-TCAGTTTGCCAGAGTTTGTTAGA-3'
	Reverse	5'-AGAGCACCAAGACTGGCTCT-3'
miR-20a-3p	Forward	5'-AACACGCACTGCATTATGAGCA-3'
	Reverse	5'- CAGTGCAGGGTCCGAGGT-3'
COL11A1	Forward	5′-TAACATCGCTGACGGGAAGTG-3′
	Reverse	5′- CCGTGATTCCATTGGTATCAACA-3′
GAPDH	Forward	5'- CTGGGCTACACTGAGCACC -3'
	Reverse	5'-AAGTGGTCGTTGAGGGCAATG-3'
U6	Forward	5′-GCTTCGGCAGCACATATACTAAAAT-3′
	Reverse	5′-CGCTTCACGAATTTGCGTGTCAT-3′
