# Supplementary material for: Circ-0005105 activates COL11A1 by targeting miR-20a-3p to promote pancreatic ductal adenocarcinoma progression
Source: Cell Death Dis. 2021 Jun 28;12(7):656. doi: 10.1038/s41419-021-03938-8 (PMC8239051; doi:10.1038/s41419-021-03938-8)
Supplement: Supplementary file 3 — supplementary table 2 [file 41419_2021_3938_MOESM3_ESM.pdf]

Supplementary Table S2. Information on antibodies used in this study

Antibody	Company
GAPDH	Proteintech Group, China
COL11A1	Abcam, USA
MMP2	Cell Signaling Technology,
MMP7	Cell Signaling Technology, USA
MMP9	Cell Signaling Technology, USA
E-cadherin	Cell Signaling Technology, USA
N-cadherin	Cell Signaling Technology, USA
β-catenin	Proteintech Group, China
Snail	Proteintech Group, China
Slug	Proteintech Group, China
Twist	Proteintech Group, China
