# Supplementary figures and images for: Circ-0005105 activates COL11A1 by targeting miR-20a-3p to promote pancreatic ductal adenocarcinoma progression
Source: Cell Death Dis. 2021 Jun 28;12(7):656. doi: 10.1038/s41419-021-03938-8 (PMC8239051; doi:10.1038/s41419-021-03938-8)

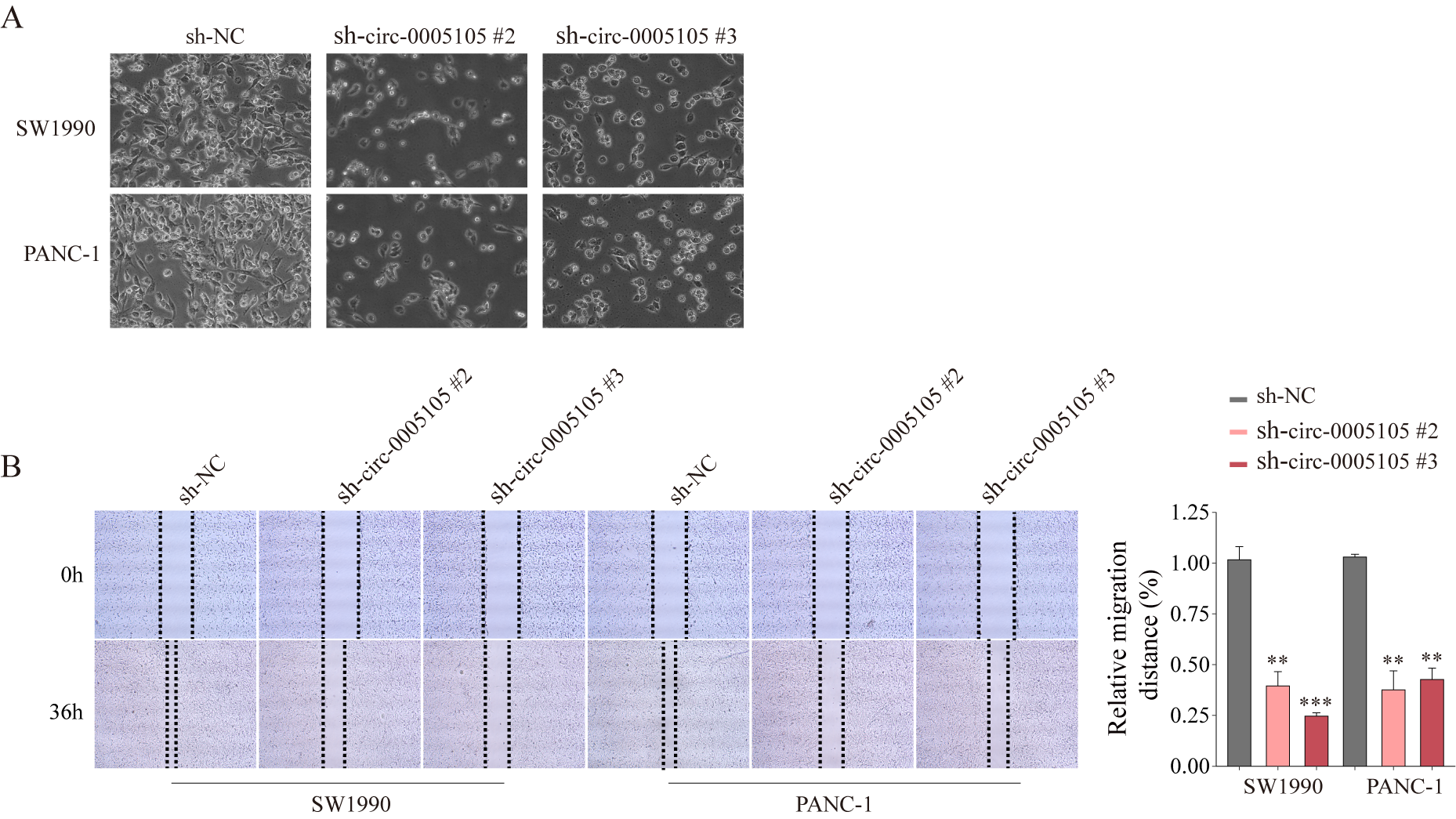

Supplement: Supplementary file 4 — Supplementary Figure S1 [file 41419_2021_3938_MOESM4_ESM.tif]

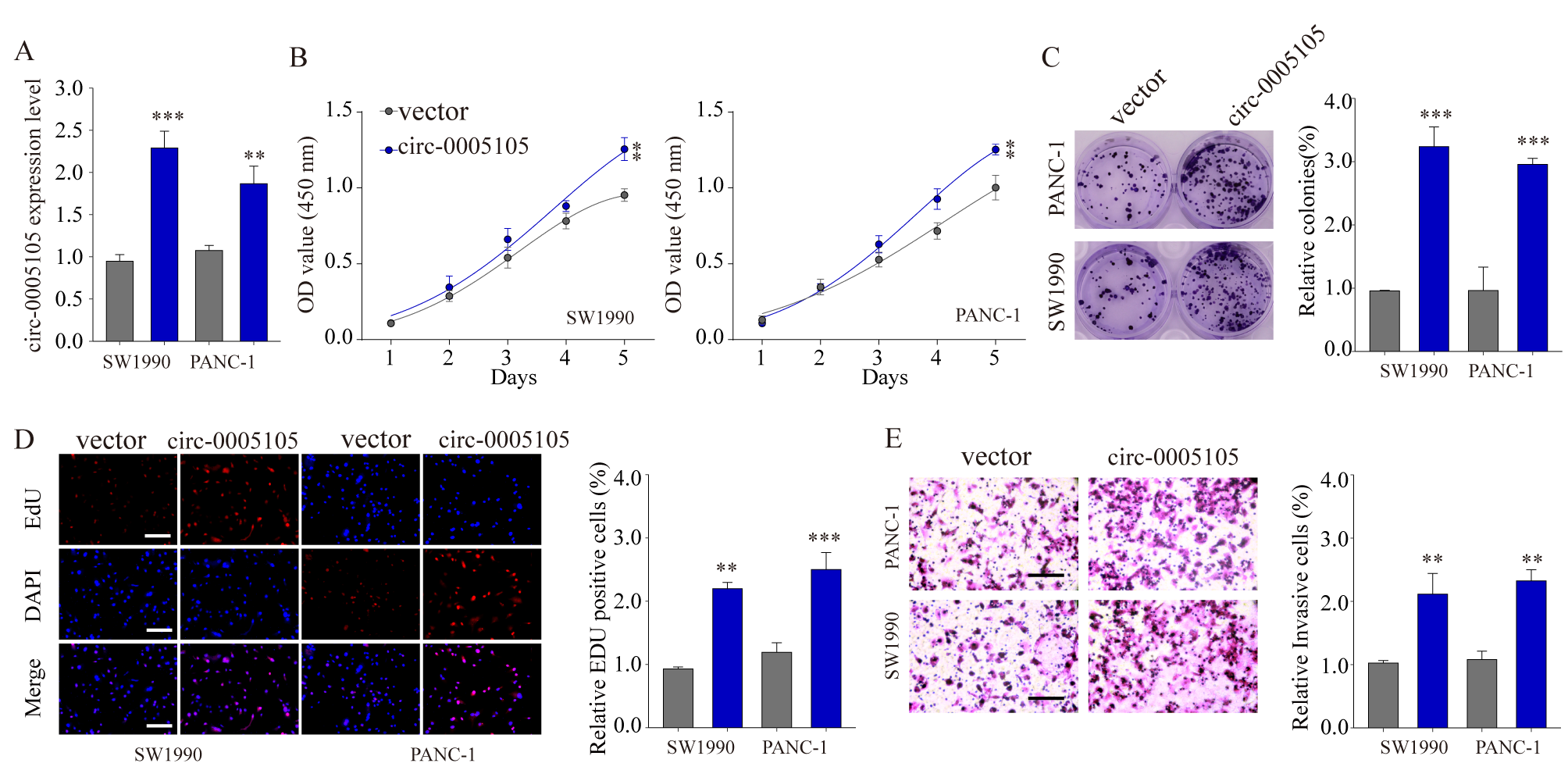

Supplement: Supplementary file 5 — Supplementary Figure S2 [file 41419_2021_3938_MOESM5_ESM.tif]

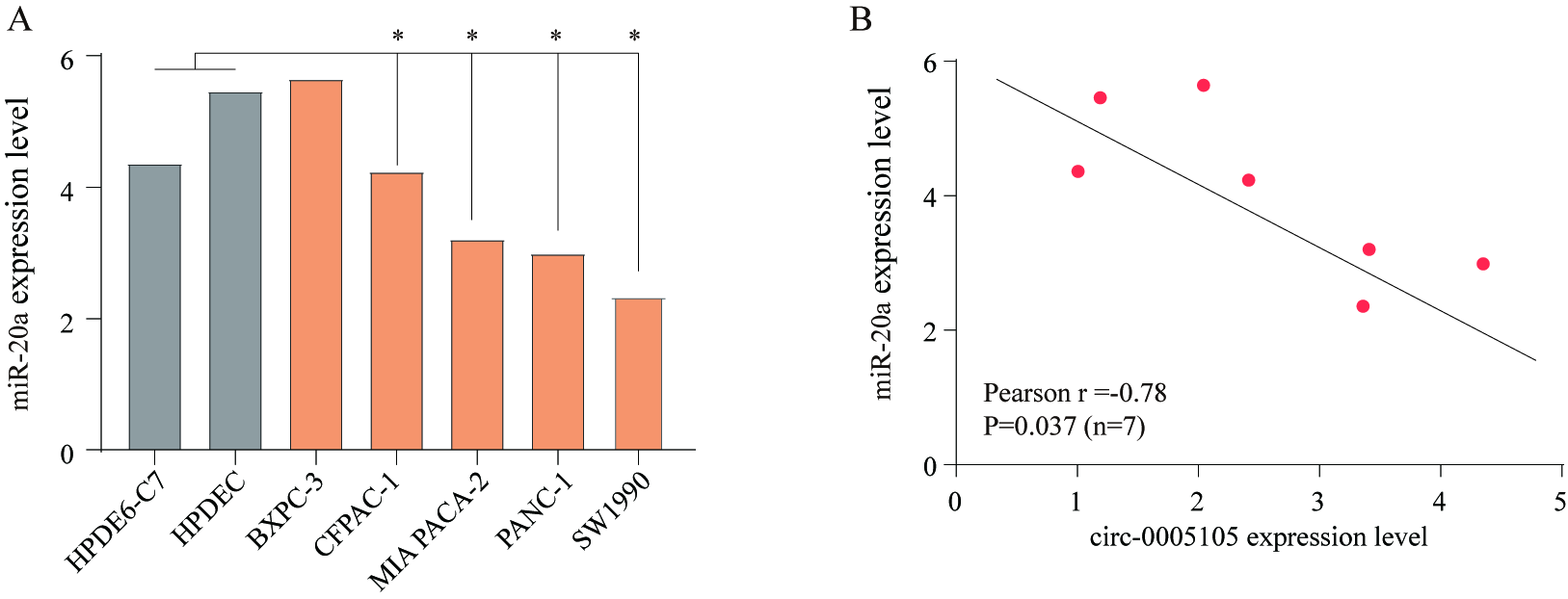

Supplement: Supplementary file 6 — Supplementary Figure S3 [file 41419_2021_3938_MOESM6_ESM.tif]

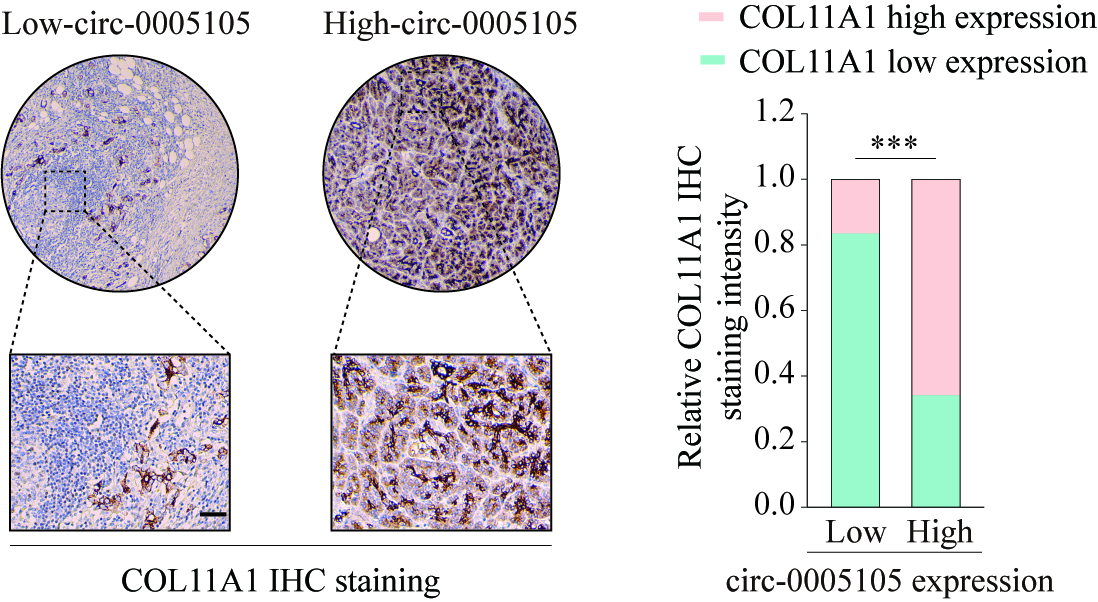

Supplement: Supplementary file 7 — Supplementary Figure S4 [file 41419_2021_3938_MOESM7_ESM.tif]

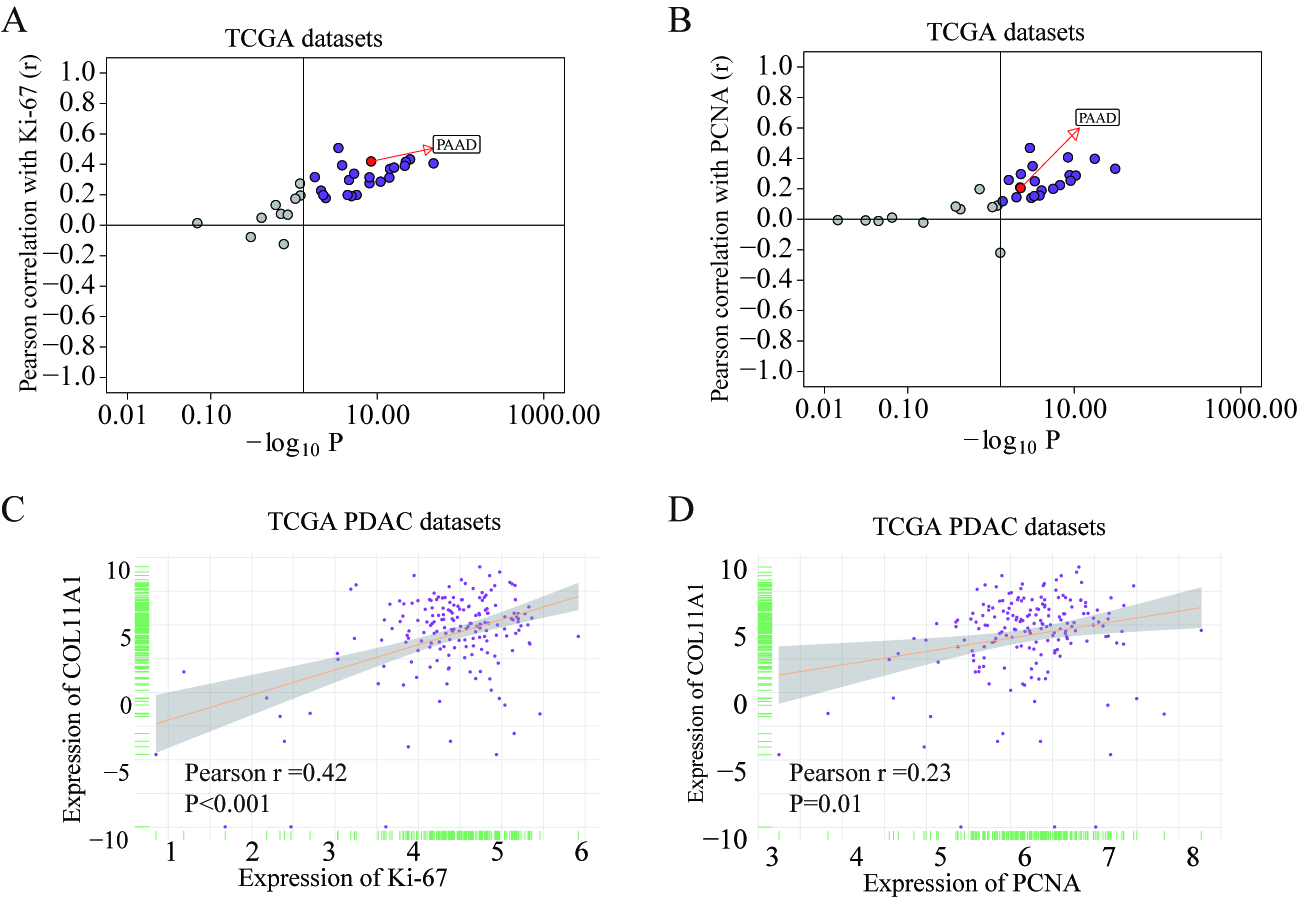

Supplement: Supplementary file 8 — Supplementary Figure S5 [file 41419_2021_3938_MOESM8_ESM.tif]

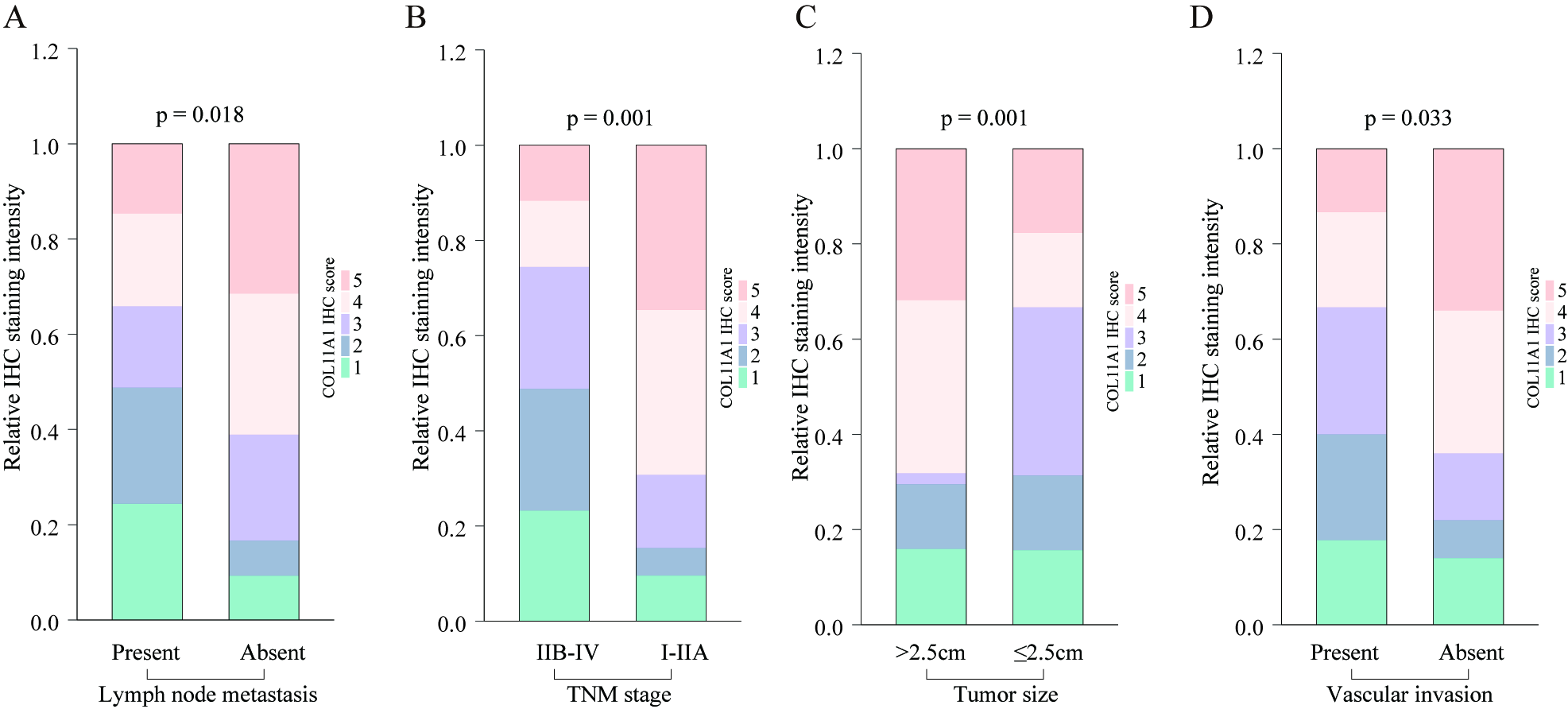

Supplement: Supplementary file 9 — Supplementary Figure S6 [file 41419_2021_3938_MOESM9_ESM.tif]

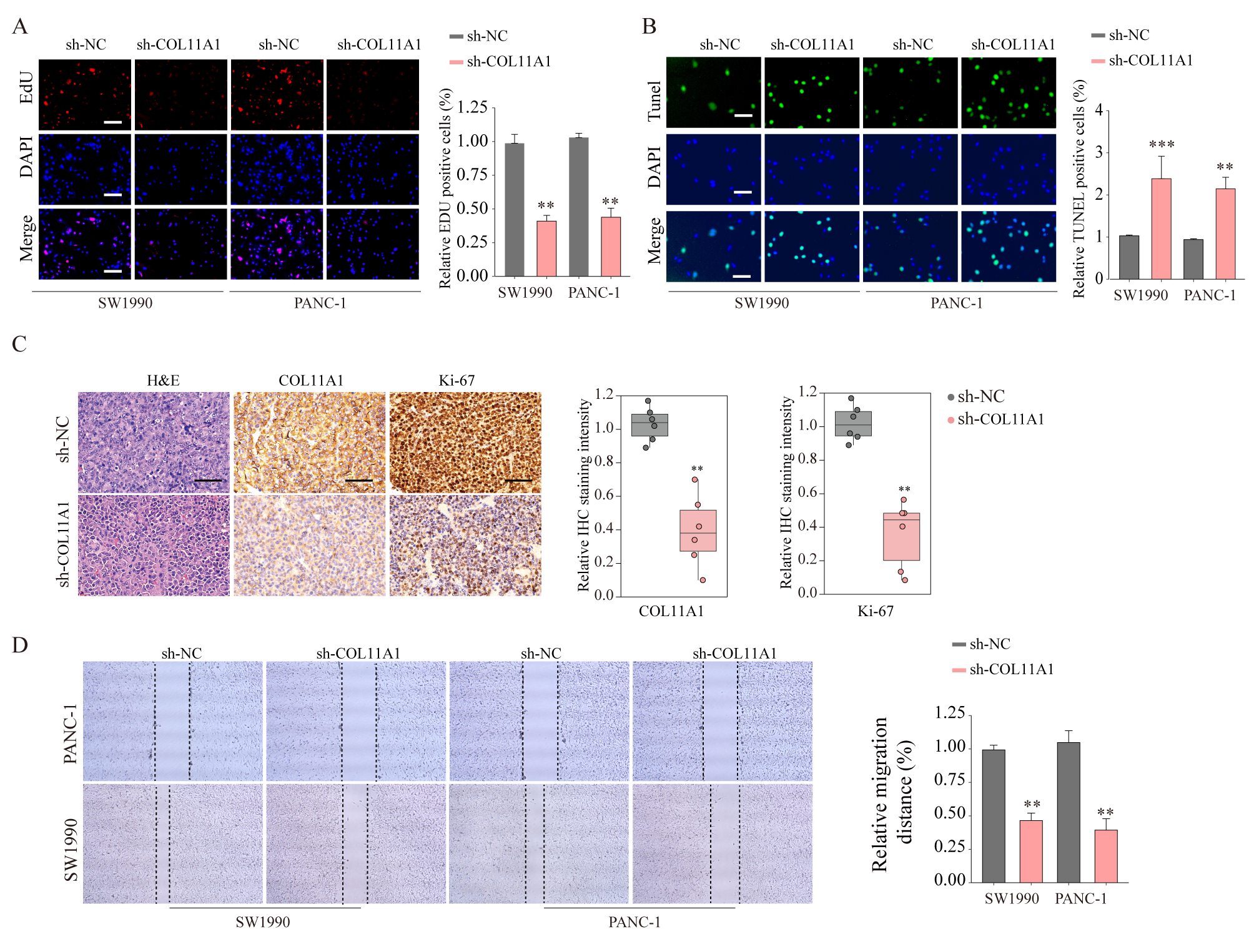

Supplement: Supplementary file 10 — Supplementary Figure S7 [file 41419_2021_3938_MOESM10_ESM.tif]

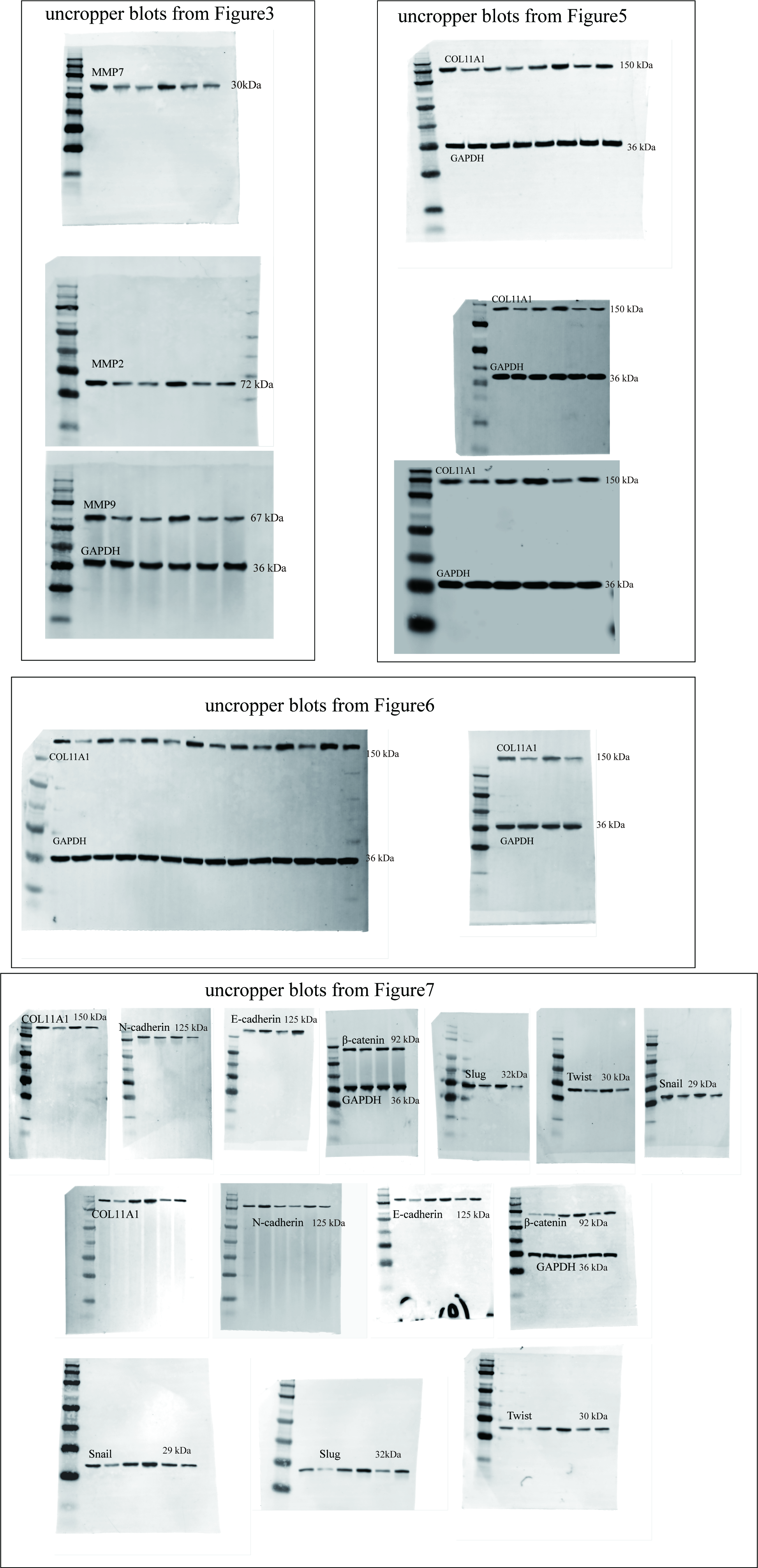

Supplement: Supplementary file 11 — Supplementary Figure S8 [file 41419_2021_3938_MOESM11_ESM.tif]
